# Supplementary material for: Promiscuity of response regulators for thioredoxin steers bacterial virulence
Source: Nat Commun. 2022 Oct 20;13:6210. doi: 10.1038/s41467-022-33983-6 (PMC9584953; doi:10.1038/s41467-022-33983-6)
Supplement: Supplementary file 1 — Supplementary Figures and Tables [file 41467_2022_33983_MOESM1_ESM.pdf]

Promiscuity of response regulators for thioredoxin steers bacterial virulence

Ju-Sim Kim[1,#], Alexandra Born[2,#], James Till[1], Lin Liu[1], Sashi Kant[1], Morkos A. Henen[2,3], Beat Vögeli[2], and Andrés Vázquez-Torres[1,3*]

[1]University of Colorado School of Medicine, Department of Immunology & Microbiology, Aurora, Colorado, USA
[2]University of Colorado School of Medicine, Department of Biochemistry & Molecular Genetics, Aurora, Colorado, USA
[3]Faculty of Pharmacy, Mansoura University, Mansoura 35516, Egypt

[4]Veterans Affairs Eastern Colorado Health Care System, Denver, Colorado, USA

[#]Equal contribution
[*]Corresponding Author: Andres.Vazquez-Torres@cuanschutz.edu,

Supplementary Figures

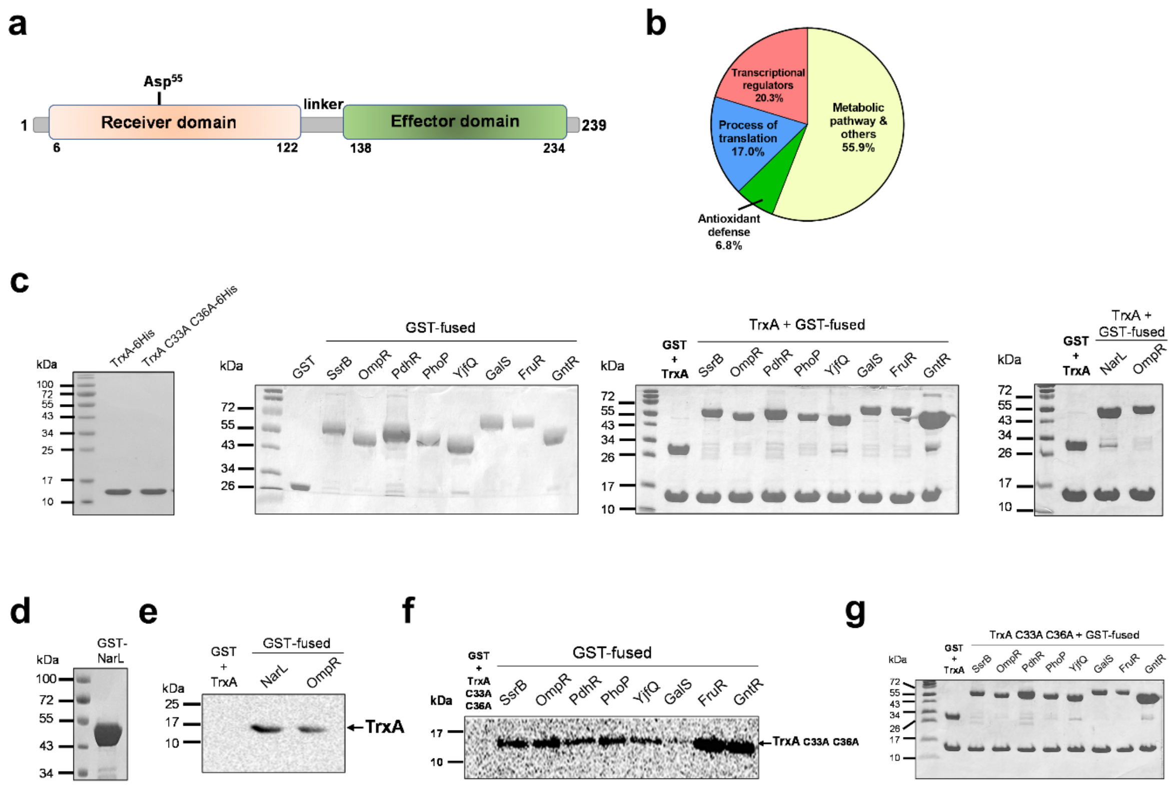

Fig S1. Thioredoxin binds to multiple transcriptional regulators. (A) Scheme of the response regulator OmpR with predicted receiver (orange box) and effector (green box) domains, the linker, and phosphorylatable Asp^{55} in the receiver domain. The total number of amino acid residues and the number of amino acids corresponding to each domain are indicated. (B) Protein classes identified by mass spectrometry to bind to *Salmonella* thioredoxin. The pie chart shows selected proteins based on peptide identification from mass spectrometry with a probability greater than 95%. (C, D and G) Purified recombinant proteins and input proteins analyzed in pull-down assays were evaluated by SDS-PAGE gels and visualized by Coomassie Brilliant Blue staining. The gels are representative of 3 independent experiments. (E) Interactions of TrxA protein (i.e., prey) with recombinant GST-NarL (i.e., bait) were determined in a biochemical pull-down assay using immunoblotting. TrxA proteins were detected bin Western blots using anti-His antibodies. GST and GST-OmpR were used as negative and positive controls, respectively. The data are representative of 3 independent experiments. (F) The binding of the TrxA C33A C36A variant lacking key cysteine residues in thioredoxin oxidoreductase catalytic domain (i.e., prey) to GST-tagged fusion recombinant proteins (i.e., bait) was assessed by biochemical pull-down assays. GST was used as a negative control. TrxA variants were probed by immunoblotting using anti-His antibodies. The data are presentative from 3 independent experiments. Source data are provided as a Source Data file.

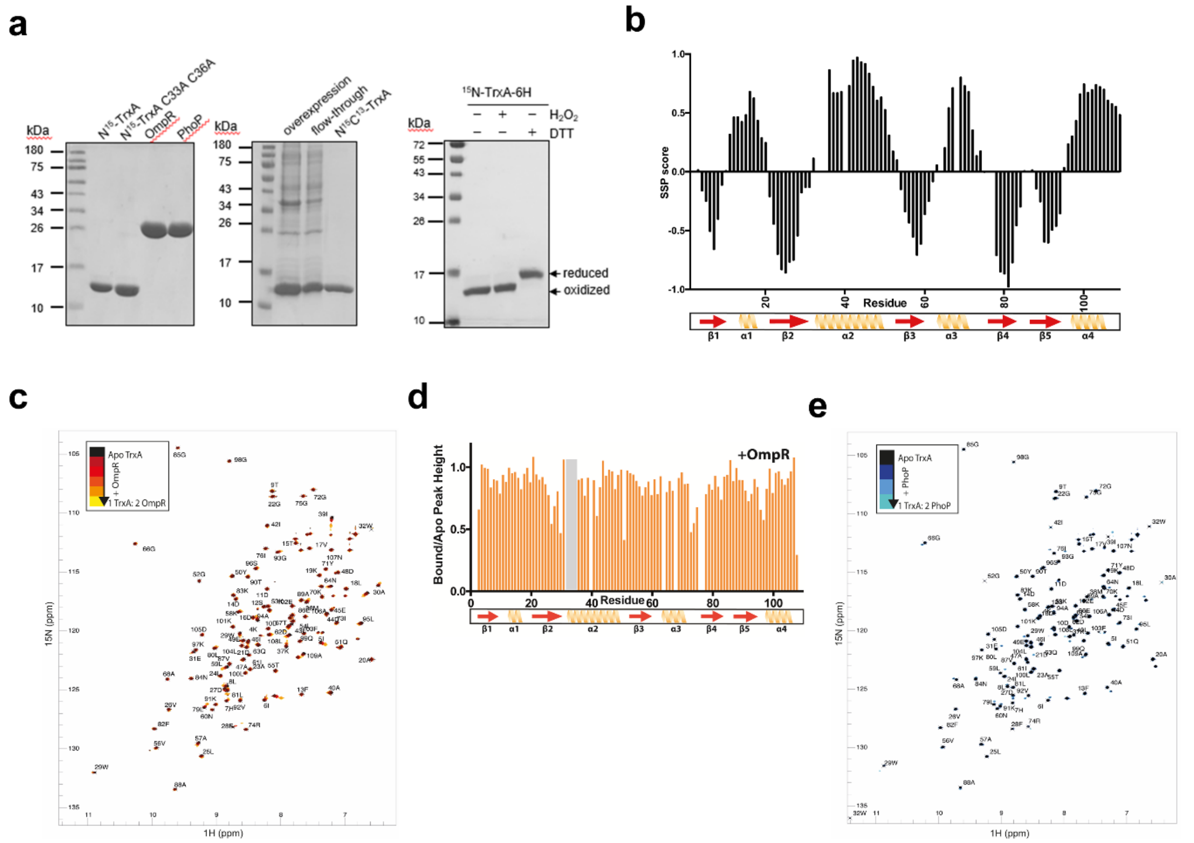

Fig S2. The TrxA binding interface is shared between OmpR and PhoP. (A) Purified ^{15}N-TrxA, ^{15}N, ^{13}C-TrxA and unlabeled OmpR and PhoP proteins were assessed on SDS-PAGE gels. Thiol redox state in cysteine residues of ^{15}N TrxA was evaluated by AMS. Proteins were treated with 1 mM H_2O_2 or 1 mM DTT at 37°C for 1 h. Specimens were visualized by Coomassie Brilliant Blue staining after separation in non-reducing SDS-PAGE. The gels are representative of 2 independent experiments. (B) Secondary structure propensity (SSP) scores for apo TrxA calculated using H^N, ^{15}N, $^{13}C\alpha$, and $^{13}C\beta$ chemical shifts for each assigned residue. A score of “+1” indicates a fully formed α-helix while “-1” indicates a β-sheet, with “0” indicating disorder. (C and E) Overlay of full ^{15}N-HSQC spectra of backbone NH resonance assigned apo TrxA (black) with increasing amounts of either unlabeled OmpR (yellow) or PhoP (blue). (D) Relative peak intensity quenching in ^{15}N-HSQC spectra of TrxA upon 1:2 titration with unlabeled OmpR. We were unable to assign the spectra for the grey region. Source data are provided as a Source Data file.

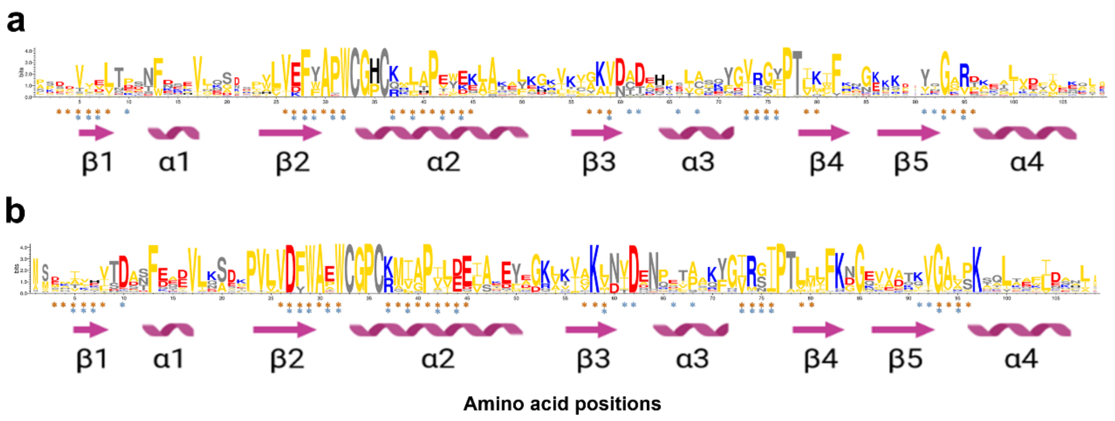

Fig. S3. Conservation of thioredoxin residues. (A) Weblogo3 representation of residue conservation across the results of a stringent PHMMER search that excluded bacterial proteins after full-length sequence alignment by Clustal Omega (*1-3*). The results include 3,184 eukaryotic, 84 archaeal, and 1 viral sequences with e-values of $\leq 10^{-23}$ with a BLOSSUM90 matrix and gap and extension penalty of 0. (B) Weblogo3 representation of residue conservation when using the HMMER results from (A) to probe only bacterial proteins after full-length sequence alignment by Clustal Omega. The bacterial results include 3,540 sequences with e-values of $\leq 10^{-32}$ when bias filtering was excluded.

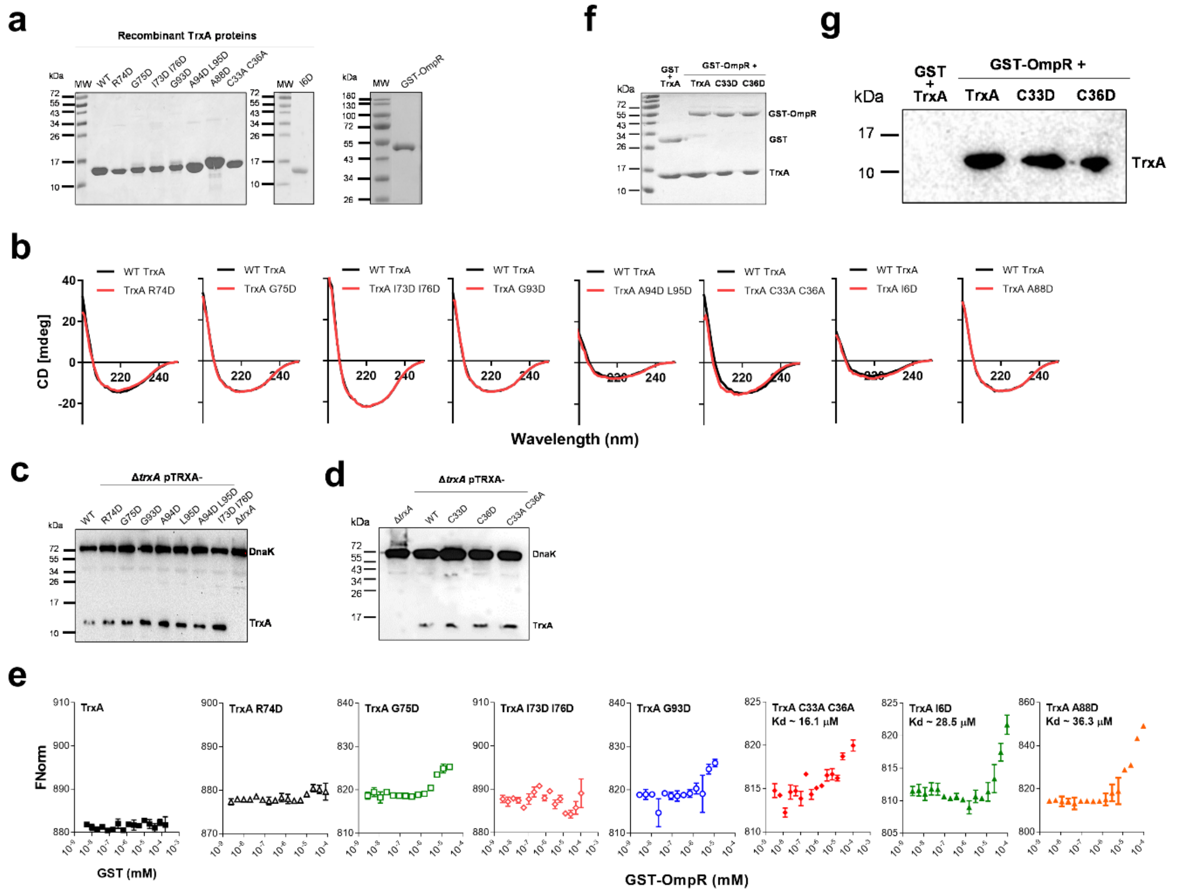

Fig. S4. Characterization of the TrxA interfacial residues that interact with OmpR. (A and F) Purified recombinant proteins and input proteins analyzed in pull-down assays were evaluated on SDS-PAGE gels and visualized by Imperial Coomassie Brilliant Blue staining. . The gels are representative of 3 independent experiments. (B) The secondary structure of TrxA variants was evaluated by CD spectroscopy. Each spectrum is the average of 3 independent scans from 2 separate experiments. (C and D) The expression of wildtype TrxA protein or its variants was assessed by immunoblotting of soluble extracts isolated from the indicated *Salmonella* strains grown overnight in LB broth. The blots are representative of 4-5 independent experiments. Expression of the housekeeping chaperone DnaK was monitored as an internal control. (E) Binding of TrxA variants to GST::OmpR was assessed by microscale thermophoresis. GST was used as a negative control. The *Kd* values that could be statistically fitted to the data are displayed. The data are the mean ± SD (n = 2 for all except n = 3 for TrxA C33A C36A) from 2 independent experiments. (G) Interactions of TrxA variants (i.e., prey) with recombinant GST-OmpR (i.e., bait) were analyzed in pull-down assays using immunoblotting. TrxA proteins were detected by immunoblotting using anti-His antibodies. GST was used as negative. The data are representative of 3 independent experiments. Source data are provided as a Source Data file.

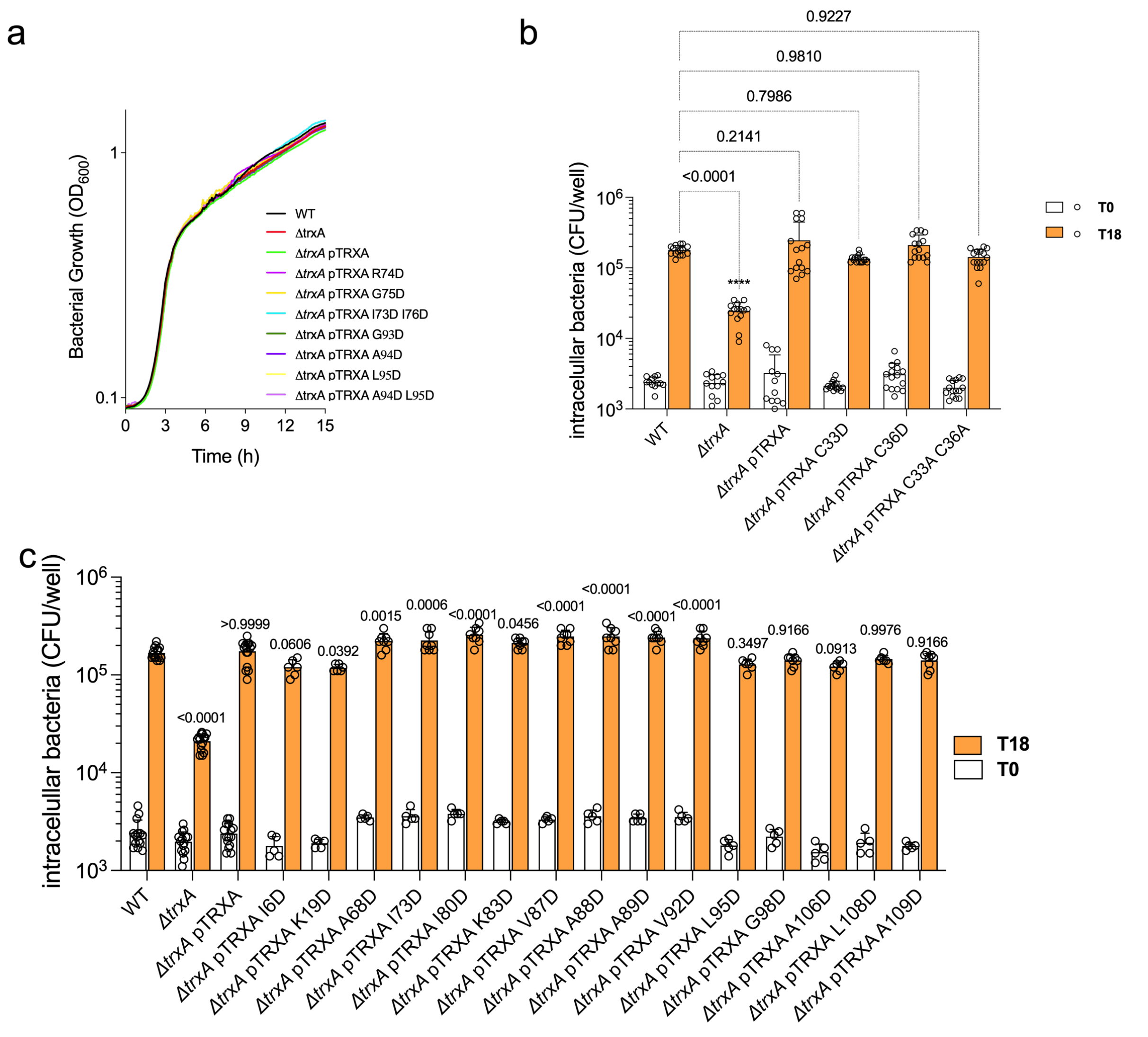

Fig. S5. Virulence of *Salmonella* strains bearing interfacial mutations in TrxA. (A) Growth of the indicated *Salmonella* strains was monitored in LB broth by measuring OD_{600} over time on a BioTek Synergy H1. Data are the mean ± SD (n = 4) from 2 independent experiments. (B and C) Intracellular replication of *Salmonella* in J774 cells 18 h post-infection was determined by CFU measurement. The number of intracellular bacteria is displayed at 0 (white column) and 18 (orange column) hours after infection. The data are the mean ± SD (B, T0, n = 12, 12, 12, 15, 15, 15; T18, n = 15, 14, 15, 15, 15, 15) (C, T0, n = 15, 15, 15, 5, 5, 5, 5, 5, 5, 5, 5, 5, 5, 5, 5, 5, 5, 5; T18, n= 15, 15, 15, 6, 6, 8, 8, 8, 8, 8, 8, 8, 8, 6, 8, 6, 6, 8) from 2-8 independent experiments. ****, $p < 0.0001$ as determined by two-way ANOVA. ns, not significant when compared to the 18 h time point of J774 cells infected with WT controls. Source data are provided as a Source Data file.

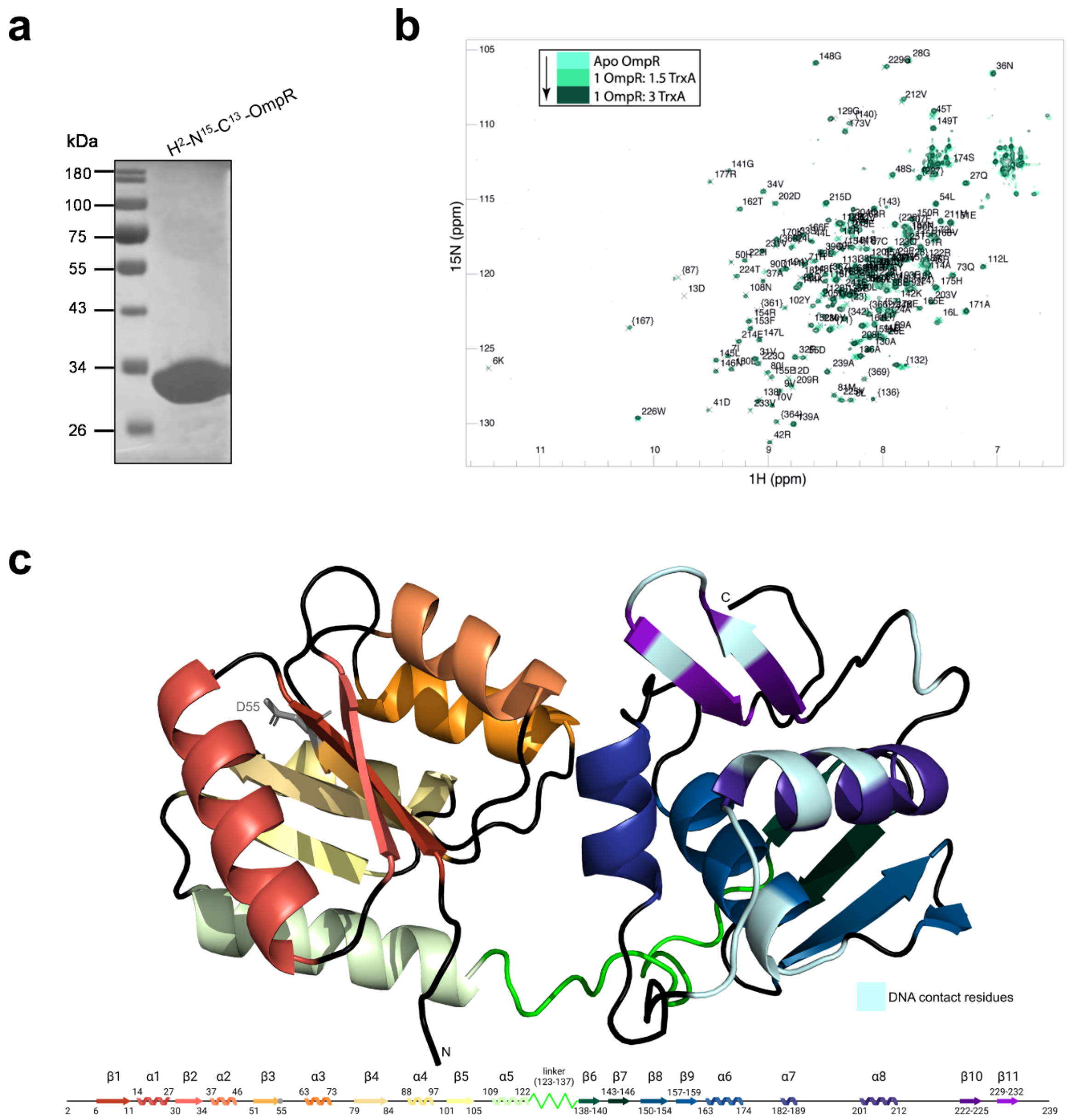

Fig. S6. Identification of residues in OmpR that mediate productive interactions with TrxA. (A) Recombinant OmpR was evaluated by SDS-PAGE gels and visualized by Coomassie Brilliant Blue staining. The gels are representative of 2 independent experiments. (B) ^{15}N-HSQC spectra of apo OmpR (cyan) with increasing amounts of TrxA (dark green). (C) An Alpha-Fold representation of OmpR (https://alphafold.ebi.ac.uk/entry/P0AA19) colored by secondary structure with the DNA-binding residues highlighted in cyan, as determined by a 5.0 Å cutoff between DNA and the C-terminal domain of OmpR seen in PDB 6LXN (https://www.rcsb.org/structure/6lxn) (*4*). Source data are provided as a Source Data file.

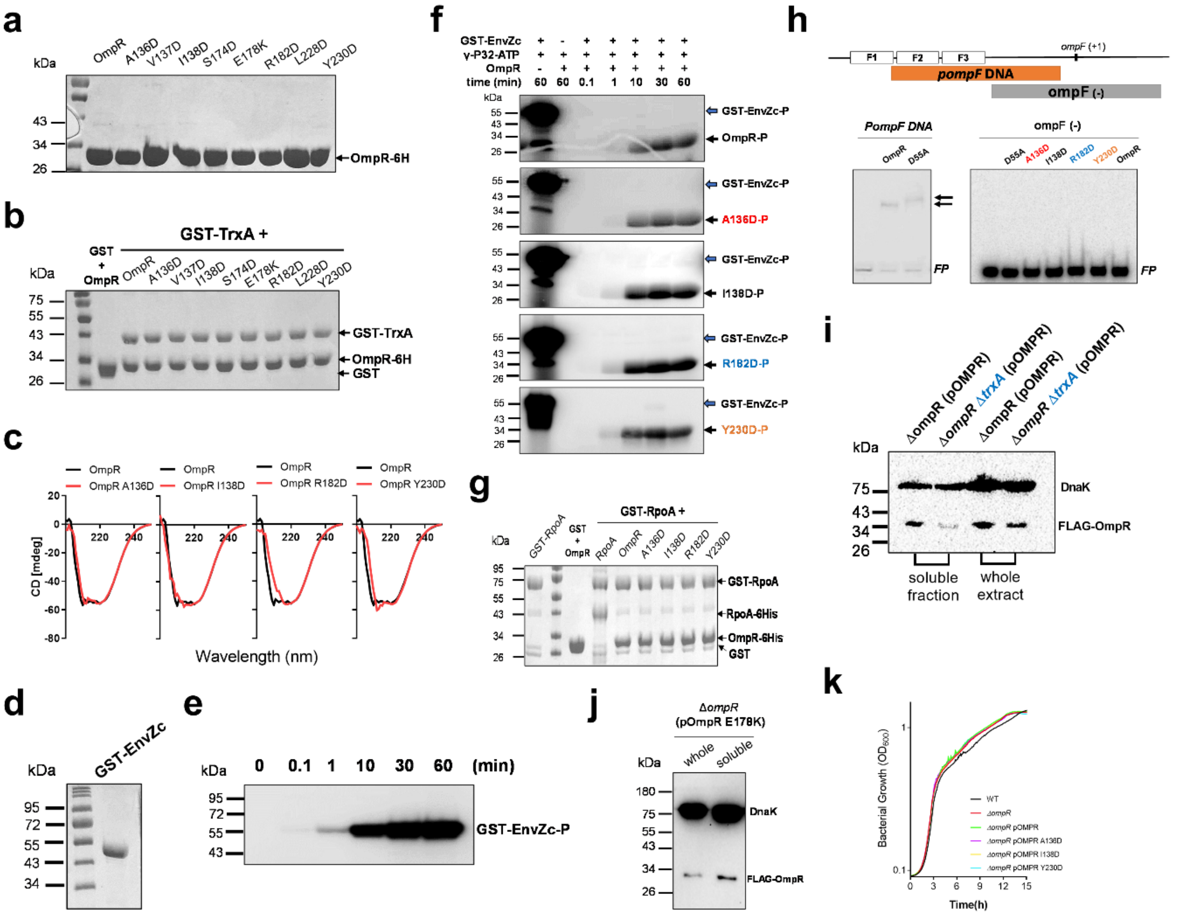

Fig. S7. Functional characterization of OmpR interfacial residues. (A and D) Purified recombinant proteins were assessed by SDS-PAGE gels and visualized by Imperial Coomassie Brilliant Blue staining. The gels are representative of 3 independent experiments. (B and G) Input proteins used in the biochemical pull-down assays were evaluated by 12% SDS PAGE gels and visualized by Imperial Coomassie Brilliant Blue staining. The gels are representative of 2 independent experiments. (C) The secondary structure of OmpR proteins was evaluated by CD spectroscopy. Each spectrum is the average of 3 independent scans from 2 independent experiments. (E) Autophosphorylation of a recombinant GST-EnvZc fragment containing residues 223-450 comprising the cytoplasmic domain of the sensor kinase EnvZ. The time-course autophosphorylation of the GST-EnvZc protein was performed in the presence of [γ-^{32}P]ATP, and the reaction products (10 μg of GST-EnvZc per lane) overtime were analyzed by a phosphorimager after separation in 10% SDS-PAGE gels. The data are representative of 3 independent experiments. (F) The time-course *in vitro* phosphorylation of recombinant OmpR variants was performed in the presence of GST-EnvZc protein prepared as in panel E. Samples were collected at the indicated times and analyzed by electrophoresis and autoradiography. The data are presentative from 3 independent experiments. (H) The top panel shows a map of the *ompF* locus showing the locations of OmpR binding sites (white boxes), as well as positive (orange box) and negative (gray box) probes used in the electrophoretic mobility shift assays. Binding of OmpR WT and D55A to the positive probe encompassing the *PompF* DNA was evaluated by electrophoretic mobility shift assays (EMSA, left panel). Representative EMSA performed with a nonspecific 80-bp DNA fragment mapping to the upstream DNA region of the *ompF* promoter was also performed (right panel). The blot is representative of 3 independent experiments. (I and J) OmpR and OmpR variants in whole-cell and soluble cytoplasmic extracts obtained from

ΔompR or *ΔtrxA ΔompR Salmonella* were visualized by immunoblots using anti-FLAG antibodies. DnaK protein was probed as an internal control. The blot is representative of 3 independent experiments. (K) Growth of the indicated *Salmonella* strains was measured in LB broth for 15 h at 37ºC by monitoring OD_{600} over time on a BioTek Synergy H1. Data are the mean ± SD (n = 4) from 2 independent experiments. Source data are provided as a Source Data file.

Supplementary Tables

Table S1. TrxA partner molecules identified by mass spectrometric analysis.

Antioxidant defense	Thioredoxin reductase (35 kDa)
	Alkyl hydroperoxide reductase (21 kDa)
	Methionine sulfoxide reductase MsrA (23 kDa)
Chaperones and translation	50S ribosomal protein L1
	60 kDa chaperon (57 kDa)
	DnaJ (40 kDa)
	DnaK (69 kDa)
	Cobalt-precorrin 2 C-methyltransferase (26 kDa)
	FKBP-type peptidyl prolyl cis-trans isomerase SlyD (21 kDa)
	Transcription termination protein NusG (21 kDa)
	Transcriptional termination factor Rho (47 kDa)
	Trigger factor (48 kDa)
	tRNA modifying protein YfgZ (36 kDa)
Metabolism	Acetyl CoA carboxylase carboxyl transferase subunit a (35 kDa)
	Acetyl CoA carboxylase carboxyl transferase subunit b (33 kDa)
	Acetyl CoA reductase (26 kDa)
	Acetyl-coenzyme A carboxylase carboxyl transferase subunit a (35 kDa)
	Acetyl-coenzyme A carboxylase carboxyl transferase subunit b (33 kDa)
	ADP-L-glycerol-D-manno-heptose-6-epimerase (35 kDa)
	Anaerobic dimethyl sulfoxide reductase (23 kDa)
	Bisphosphate nucleotase CycQ (27 kDa)
	Fe/S biogenesis protein NfuA (21 kDa)

	Fructose-1,6,-biophosphtase class I (37 kDa)
	Fumarate hydratase class I (64 kDa)
	Fumarate reductase iron-sulfer subunit (27 kDa)
	Glyceraldehyde -3-phosphate dehydrogenase (36 kDa)
	GTP binding protein EngB (24 kDa)
	Malate dehydrogenase (32 kDa)
	Outer membrane protein A OmpA (37 kDa)
	Outer membrane protein D OmpD (40 kDa)
	Outer membrane protein F OmpF (39 kDa)
	PhoH like protein (39 kDa),
	Phosphoadenosine phosphosulfate reductase (28 kDa)
	Pyrroline-5-carboxylate reductase (28 kDa)
	Riboflavin synthase (23 kDa)
	Ribose-phosphate pyrophosphokinase (34 kDa)
	Succinate dehydrogenase iron-sulfur subunit (27 kDa)
	Thiosulfate reductase electron transport protein PhsB (21 kDa)
	UDP-3-O-glucosamin N-acyltransferase (36 kDa)
	Uridine phosphorylase (27 kDa)
Transcription factors	cAMP activated global transcriptional regulator CRP (24 kDa)
	Catabolite repressor/activator (38 kDa),<br>encoded by genes *cra* (also known as *fruR*)
	HTH (helix-turn-helix)-type transcriptional regulator AIIR (29 kDa)
	HTH-type transcriptional regulator CysB (36 kDa)
	HTH-type transcriptional regulator UlaR (27 kDa),<br>encoded by the *yjfQ* gene
	HTH-type transcriptional repressor FabR (24 kDa)
	HTH-type transcriptional regulator GalS (39 kDa)
	HTH-type transcriptional regulator GntR (36 kDa)

	Pyruvate dehydrogenase repressor PdhR (29 kDa)
	Response regulator OmpR (27 kDa)
	Response regulator PhoP (26 kDa)
	Transcriptional regulator LsrR (34 kDa)

Table S2. Bacteria used in this study.

Strains	Relevant characteristics	Reference
Salmonella		
14028s	wild type of *S. enterica* serovar *Typhimurium*	ATCC
AV19007	Δ*ompR envZ*::Km [Δ*ompR*]	This study
AV19021	Δ*ompR envZ*::Km (pFLAG::OMPR-ENVZ [pOMPR])	This study
AV21093	Δ*ompR envZ*::Km (pFLAG::OMPR A136D-ENVZ [pOMPR A136D])	This study
AV21095	Δ*ompR envZ*::Km (pFLAG::OMPR I138D-ENVZ [pOMPR I138D])	This study
AV21096	Δ*ompR envZ*::Km (pFLAG::OMPR E178K-ENVZ [pOMPR E178K])	This study
AV21097	Δ*ompR envZ*::Km (pFLAG::OMPR R182D-ENVZ [pOMPR R182D])	This study
AV21098	Δ*ompR envZ*::Km (pFLAG::OMPR Y230D-ENVZ [pOMPR Y230D])	This study
AV09137	Δ*trxA*::FRT	(*5*)
AV20119	Δ*trxA*::FRT (pWSK29::TAP)	This study
AV10195	Δ*trxA*::FRT (pWSK29::*trxA*::TAP)	(*5*)
AV18058	Δ*trxA*::FRT (pTRXA::FLAG)	This study
AV18061	Δ*trxA*::FRT (pTRXA::FLAG R74D)	This study
AV19196	Δ*trxA*::FRT (pTRXA::FLAG G75D)	This study
AV20057	Δ*trxA*::FRT (pTRXA::FLAG I73D I76D)	This study
AV19197	Δ*trxA*::FRT (pTRXA::FLAG G93D)	This study
AV18112	Δ*trxA*::FRT (pTRXA::FLAG A94D)	This study
AV18078	Δ*trxA*::FRT (pTRXA::FLAG L95D)	This study
AV21154	Δ*trxA*::FRT (pTRXA::FLAG A94D L95D)	This study
AV19027	Δ*trxA*::FRT, Δ*ompR envZ*::Km	This study
AV19033	Δ*trxA*::FRT, Δ*ompR envZ*::Km (pFLAG::OMPR-ENVZ)	This study
AV22055	FLAG::*ompR envZ*::Cm	This study
AV22053	*trxA::6His*::Km	This study
AV22070	FLAG::*ompR envZ*::Cm*, trxA::6His*::Km	This study
E.coli		
DH5α	*supE*44 Δ*lacU*169 (ϕ80 *lacZ* ΔM15) *hsdR*17 *recA*1 *endA*1 *gyrA*96 *thi*-1 *relA*1	(*6*)
BTH101	F- *cya*-99 *ara*D139 *gal*E15 *gal*K16 *rps*L1 (Str^r) *hsd*R2 *mcr*A1 *mcr*B1	Euromedex
BL21(DE3)	F$^-$ *ompT hsd*S$_B$(r$_B^-$ m$_B^-$) *gal dcm* (DE3)	Invitrogen
OrigamiB(DE3)	F$^-$ *ompT hsd*S$_B$(r$_B^-$ m$_B^-$) *gal dcm lacY1 ahpC* (DE3) *gor522*::Tn*10 trxB* pLysS (Cam^R, Kan^R, Tet^R)	Novagen
AV16097	BTH101 (pKNT25::*fruR,* pUT18*::trxA*)	This study
AV16108	BTH101 (pKNT25::*fruR,* pUT18*::trxA* C33A C36A)	This study
AV16096	BTH101 (pKNT25::*galS,* pUT18*::trxA*)	This study
AV16107	BTH101 (pKNT25::*galS,* pUT18*::trxA* C33A C36A)	This study
AV16098	BTH101 (pKNT25::*gntR,* pUT18*::trxA*)	This study
AV16109	BTH101 (pKNT25::*gntR,* pUT18*::trxA* C33A C36A)	This study
AV16090	BTH101 (pKNT25::*ompR,* pUT18*::trxA*)	This study
AV16104	BTH101 (pKNT25::*ompR,* pUT18*::trxA* C33A C36A)	This study
AV16112	BTH101 (pKNT25::*phoP,* pUT18*::trxA*)	This study
AV16115	BTH101 (pKNT25::*phoP,* pUT18*::trxA* C33A C36A)	This study
AV21152	BTH101 (pKNT25::*pdhR,* pUT18*::trxA*)	This study
AV21153	BTH101 (pKNT25::*pdhR,* pUT18*::trxA* C33A C36A)	This study
AV12160	BTH101 (pKNT25::*ssrB,* pUT18*::trxA*)	(*5*)

AV12156	BTH101 (pKNT25::*ssrB,* pUT18*::trxA* C33A C36A)	(*5*)
AV16091	BTH101 (pKNT25::*yjfQ,* pUT18*::trxA*)	This study
AV16105	BTH101 (pKNT25::*yjfQ,* pUT18*::trxA* C33A C36A)	This study
AV19178	BTH101 (pKT25*,* pUT18C*::trxA*)	This study
AV20117	BTH101 (pKT25::*ompR,* pUT18C)	This study
AV19183	BTH101 (pKT25::*ompR,* pUT18C*::trxA*)	This study
AV19216	BTH101 (pKT25::*ompR,* pUT18C*::trxA* R74D)	This study
AV19217	BTH101 (pKT25::*ompR,* pUT18C*::trxA* G75D)	This study
AV19218	BTH101 (pKT25::*ompR,* pUT18C*::trxA* G93D)	This study
AV19219	BTH101 (pKT25::*ompR,* pUT18C*::trxA* A94D L95D)	This study
AV20118	BTH101 (pKT25::*phoP,* pUT18C)	This study
AV19184	BTH101 (pKT25::*phoP,* pUT18C*::trxA*)	This study
AV19222	BTH101 (pKT25::*phoP,* pUT18C*::trxA* R74D)	This study
AV19223	BTH101 (pKT25::*phoP,* pUT18C*::trxA* G75D)	This study
AV19224	BTH101 (pKT25::*phoP,* pUT18C*::trxA* G93D)	This study
AV19225	BTH101 (pKT25::*phoP,* pUT18C*::trxA* A94D L95D)	This study
AV19182	BTH101 (pKT25::*ssrB,* pUT18C)	This study
AV19179	BTH101 (pKT25::*ssrB,* pUT18C*::trxA*)	This study
AV19213	BTH101 (pKT25::*ssrB,* pUT18C*::trxA* R74D)	This study
AV19199	BTH101 (pKT25::*ssrB,* pUT18C*::trxA* G75D)	This study
AV19200	BTH101 (pKT25::*ssrB,* pUT18C*::trxA* G93D)	This study
AV19201	BTH101 (pKT25::*ssrB,* pUT18C*::trxA* A94D L95D)	This study
AV21123	BTH101 (pKT25::*ompR* A136D*,* pUT18C*::trxA*)	This study
AV21124	BTH101 (pKT25::*ompR* I138D*,* pUT18C*::trxA*)	This study
AV21125	BTH101 (pKT25:: *ompR* R182D*,* pUT18C*::trxA*)	This study
AV21126	BTH101 (pKT25:: *ompR* Y230D*,* pUT18C*::trxA*)	This study
AV15104	BTH101 (pKT25::*rpoA,* pUT18C*::trxA*)	This study
AV15128	BTH101 (pKT25::*rpoA,* pUT18C*::trxA* C33A C36A)	This study
AV21066	BL21(DE3) (pET22b::OmpR A136D)	This study
AV21067	BL21(DE3) (pET22b::OmpR V137D)	This study
AV21071	BL21(DE3) (pET22b::OmpR I138D)	This study
AV21068	BL21(DE3) (pET22b::OmpR S174D)	This study
AV21069	BL21(DE3) (pET22b::OmpR E178K)	This study
AV21070	BL21(DE3) (pET22b::OmpR R182D)	This study
AV21076	BL21(DE3) (pET22b::OmpR L228D)	This study
AV20003	BL21(DE3) (pET22b::PhoP)	This study
AV20115	BL21(DE3) (pET22b::TrxA)	This study
AV20116	BL21(DE3) (pET22b::TrxA C33A C36A)	This study
AV22105	BL21(DE3) (pET22b::TrxA C33D)	This study
AV22097	BL21(DE3) (pET22b::TrxA C36D)	This study
AV18136	BL21(DE3) (pET22b::TrxA R74D)	This study
AV19249	BL21(DE3) (pET22b::TrxA G75D)	This study
AV20089	BL21(DE3) (pET22b::TrxA I73D I76D)	This study
AV20021	BL21(DE3) (pET22b::TrxA A88D)	This study
AV19240	BL21(DE3) (pET22b::TrxA G93D)	This study
AV18141	BL21(DE3) (pET22b::TrxA A94D L95D)	This study
AV22127	BL21(DE3) (pGEX6p::EnvZc)	This study
AV16141	BL21(DE3) (pGEX6p::FruR)	This study
AV16140	BL21(DE3) (pGEX6p::GalS)	This study
AV16137	BL21(DE3) (pGEX6p::GntR)	This study
AV19006	BL21(DE3) (pGEX6p::NarL)	This study
AV16138	BL21(DE3) (pGEX6p::OmpR)	This study
AV16155	BL21(DE3) (pGEX6p::PhoP)	This study

AV16139	BL21(DE3) (pGEX6p::PdhR)	This study
AV19188	BL21(DE3) (pGEX6p::SsrB)	This study
AV07260	BL21(DE3) (pGEX6p::SsrBc)	(*7*)
AV09166	BL21(DE3) (pGEX6p::TrxA)	This study
AV16156	BL21(DE3) (pGEX6p::YjfQ)	This study
AV21132	DH5α (pTim::*ompF*)	This study
AV20008	Origami B(DE3) pLysS (pET22b::OmpR)	This study
AV20012	Origami B(DE3) pLysS (pET22b::OmpR D55A)	This study
AV21074	Origami B(DE3) pLysS (pET22b::OmpR Y230D)	This study
AV15158	Origami B(DE3) pLysS (pET22b::RpoA)	This study
AV15152	Origami B(DE3) pLysS (pGEX6p)	(*8*)
AV15155	Origami B(DE3) pLysS (pGEX6p::RpoA)	This study

Table S3. Plasmids used in this study.

Plasmid	Relevant characteristics	Source
pET22b(+)	*ori* pBR322, C-terminal 6His·Taq fusion vector, Pn^r	Novagen
pET22b::OmpR	pET-22b(+) + 0.72-kb *ompR* DNA, Pn^r	This study
pET22b::OmpR D55A	pET-22b(+) + 0.72-kb *ompR* D55A DNA, Pn^r	This study
pET22b::OmpR A136D	pET-22b(+) + 0.72-kb *ompR* A136D DNA, Pn^r	This study
pET22b::OmpR V137D	pET-22b(+) + 0.72-kb *ompR* V137D DNA, Pn^r	This study
pET22b::OmpR I138D	pET-22b(+) + 0.72-kb *ompR* I138D DNA, Pn^r	This study
pET22b::OmpR S174D	pET-22b(+) + 0.72-kb *ompR* S174D DNA, Pn^r	This study
pET22b::OmpR E178K	pET-22b(+) + 0.72-kb *ompR* E178K DNA, Pn^r	This study
pET22b::OmpR R182D	pET-22b(+) + 0.72-kb *ompR* R182D DNA, Pn^r	This study
pET22b::OmpR L228D	pET-22b(+) + 0.72-kb *ompR* L228D DNA, Pn^r	This study
pET22b::OmpR Y230D	pET-22b(+) + 0.72-kb *ompR* Y230D DNA, Pn^r	This study
pET22b::PhoP	pET-22b(+) + 0.67-kb *phoP* DNA, Pn^r	This study
pET22b::RpoA	pET-22b(+) + 0.99-kb *rpoA* DNA, Pn^r	This study
pET22b::TrxA	pET-22b(+) + 0.33-kb *trxA* DNA, Pn^r	(*5*)
pET22b::TrxA C33A C36A	pET-22b(+) + 0.33-kb *trxA* C33A C36A DNA, Pn^r	(*5*)
pET22b::TrxA C33D	pET-22b(+) + 0.33-kb *trxA* C33D DNA, Pn^r	This study
pET22b::TrxA C36D	pET-22b(+) + 0.33-kb *trxA* C36D DNA, Pn^r	This study
pET22b::TrxA R74D	pET-22b(+) + 0.33-kb *trxA* R74D DNA, Pn^r	This study
pET22b::TrxA G75D	pET-22b(+) + 0.33-kb *trxA* G75D DNA, Pn^r	This study
pET22b::TrxA I73D I76D	pET-22b(+) + 0.33-kb *trxA* I73D I76D DNA, Pn^r	This study
pET22b::TrxA A88D	pET-22b(+) + 0.33-kb *trxA* A88D DNA, Pn^r	This study
pET22b::TrxA G93D	pET-22b(+) + 0.33-kb *trxA* G93D DNA, Pn^r	This study
pET22b::TrxA A94D	pET-22b(+) + 0.33-kb *trxA* A94D DNA, Pn^r	This study
pET22b::TrxA A94D L95D	pET-22b(+) + 0.33-kb *trxA* A94D L95D DNA, Pn^r	This study
pGEX6p	GST fusion expression vector, Pn^r	Cytiva
pGEX6p::EnvZc	pGEX6p + 0.70-kb *EnvZc* DNA, Pn^r	This study
pGEX6p::FruR	pGEX6p + 1.00-kb *fruR* DNA, Pn^r	This study
pGEX6p::GalS	pGEX6p + 1.04-kb *galS* DNA, Pn^r	This study
pGEX6p::GntR	pGEX6p + 0.91-kb *gntR* DNA, Pn^r	This study
pGEX6p::NarL	pGEX6p + 0.65-kb *narL* DNA, Pn^r	This study
pGEX6p::OmpR	pGEX6p + 0.72-kb *ompR* DNA, Pn^r	This study
pGEX6p::PhoP	pGEX6p + 0.67-kb *phoP* DNA, Pn^r	This study
pGEX6p::PdhR	pGEX6p + 0.76-kb *pdhR* DNA, Pn^r	This study
pGEX6p::RpoA	pGEX6p + 0.99-kb *rpoA* DNA, Pn^r	This study
pGEX6p::SsrB	pGEX6p + 0.64-kb *ssrB* DNA, Pn^r	This study
pGEX6p::SsrBc	pGEX6p + 0.23-kb C-terminal of *ssrB* DNA (137-212 aa), Pn^r	(*7*)

pGEX6p::TrxA	pGEX6p + 0.33-kb *trxA* DNA, Pn^r	This study
pGEX6p::YjfQ	pGEX6p + 0.76-kb *yjfQ* DNA, Pn^r	This study
pKD13	template vector for FRT-flanked Km^r cassette, Km^r Pn^r	(*9*)
pKNT25	pSU40 with the N-terminal T25 domain of CyaA, Km^r	Euromedex
pKT25	pSU40 with the C-terminal T25 domain of CyaA, Km^r	Euromedex
pKNT25::FruR	pKT25 plasmid with *cyaAT25-fruR* fusion, Km^r	This study
pKNT25::GalS	pKT25 plasmid with *cyaAT25-galS* fusion, Km^r	This study
pKNT25::GntR	pKT25 plasmid with *cyaAT25-gntR* fusion, Km^r	This study
pKNT25::OmpR	pKT25 plasmid with *cyaAT25-ompR* fusion, Km^r	This study
pKNT25::PhoP	pKT25 plasmid with *cyaAT25-phoP* fusion, Km^r	This study
pKNT25::PdhR	pKT25 plasmid with *cyaAT25-pdhR* fusion, Km^r	This study
pKNT25::SsrB	pKT25 plasmid with *cyaAT25-ssrB* fusion, Km^r	(*5*)
pKNT25::YjfQ	pKT25 plasmid with *cyaAT25-yjfQ* fusion, Km^r	This study
pKT25::OmpR	pKT25 plasmid with *ompR-cyaAT25* fusion, Km^r	This study
pKT25::PhoP	pKT25 plasmid with *phoP-cyaAT25* fusion, Km^r	This study
pKT25::SsrB	pKT25 plasmid with *ssrB-cyaAT25* fusion, Km^r	This study
pKT25::RpoA	pKT25 plasmid with *rpoA-cyaAT25* fusion, Km^r	This study
pTIM	*in vitro* transcription backbone plasmid, bla *rrnB* & *rpoC* term pBluescript, Pn^r	(*10*)
pTIM::*ompF*	pTim + 1.3-kb p*ompF* and *ompF* DNA, Pn^r	This study
pUT18	pUC19 with the N-terminal T18 domain of CyaA, Pn^r	Euromedex
pUT18C	pUC19 with the C-terminal T18 domain of CyaA, Pn^r	Euromedex
pUT18::*trxA*	pUT18 plasmid with *cyaAT18-trxA* fusion, Pn^r	(*5*)
pUT18::*trxA* C33A C36A	pUT18 plasmid with *cyaAT18-trxA* C33A C36A fusion, Pn^r	(*5*)
pUT18C::*trxA*	pUT18 plasmid with *trxA-cyaAT18* fusion, Pn^r	This study
pUT18C:: *trxA* C33A C36A	pUT18 plasmid with *trxA* C33A C36A-*cyaAT18* fusion, Pn^r	This study
pUT18C:: *trxA* R74D	pUT18 plasmid with *trxA* R74D-*cyaAT18* fusion, Pn^r	This study
pUT18C:: *trxA* G75D	pUT18 plasmid with *trxA* G75D-*cyaAT18* fusion, Pn^r	This study
pUT18C:: *trxA* G93D	pUT18 plasmid with *trxA* G93D-*cyaAT18* fusion, Pn^r	This study
pUT18C:: *trxA* A94D L95D	pUT18 plasmid with *trxA* A94D L95D-*cyaAT18* fusion, Pn^r	This study
pWSK29	low copy plasmid, *lacZ*α, Pn^r	(*11*)
pWSK29::TAP	pWSK29 + 0.42-kb TAP DNA, Pn^r	(*5*)
pWSK29::*trxA*::TAP	pWSK29 + 0.90-kb p*trxA, trxA*, and TAP DNA, Pn^r	(*5*)
pFLAG::OMPR ENVZ	pWSK29 + 2.3-kb p*ompR::*FLAG*::ompR envZ* DNA, Pn^r	This study
pFLAG::OMPRA136D ENVZ	pWSK29 + 2.3-kb p*ompR::*FLAG*::ompR*A136D *envZ* DNA, Pn^r	This study

pFLAG::OMPRI138D ENVZ	pWSK29 + 2.3-kb p*ompR::*FLAG*::ompR*I138D *envZ* DNA, Pn^{r}	This study
pFLAG::OMPRE178K ENVZ	pWSK29 + 2.3-kb p*ompR::*FLAG*::ompR*IE178K *envZ* DNA, Pn^{r}	This study
pFLAG::OMPRR182D ENVZ	pWSK29 + 2.3-kb p*ompR::*FLAG*::ompR*R182D *envZ* DNA, Pn^{r}	This study
pFLAG::OMPRY230D ENVZ	pWSK29 + 2.3-kb p*ompR::*FLAG*::ompR*Y230D *envZ* DNA, Pn^{r}	This study
pTRXA::FLAG	pWSK29 + 0.51-kb p*trxA::trxA::*FLAG DNA, Pn^{r}	This study
pTRXA R74D::FLAG	pWSK29 + 0.51-kb p*trxA::trxA* R74D*::*FLAG DNA, Pn^{r}	This study
pTRXA G75D::FLAG	pWSK29 + 0.51-kb p*trxA::trxA* G75D*::*FLAG DNA, Pn^{r}	This study
pTRXA I73D I76D::FLAG	pWSK29 + 0.51-kb p*trxA::trxA* I73D I76D*::*FLAG DNA, Pn^{r}	This study
pTRXA G93D::FLAG	pWSK29 + 0.51-kb p*trxA::trxA* G93D*::*FLAG DNA, Pn^{r}	This study
pTRXA A94D::FLAG	pWSK29 + 0.51-kb p*trxA::trxA* A94D*::*FLAG DNA, Pn^{r}	This study
pTRXA L95D::FLAG	pWSK29 + 0.51-kb p*trxA::trxA* L95D*::*FLAG DNA, Pn^{r}	This study
pTRXA A94D L95D::FLAG	pWSK29 + 0.51-kb p*trxA::trxA* A94D L95D*::*FLAG DNA, Pn^{r}	This study

Table S4. Oligonucleotides used in this study.

Strains	Primer Sequence (5’ → 3’)
Δ*ompR envZ*::Km	**F:** ATAAGATTCTGGTGGTTGATGACGATATGCGTCTGCGGGTGT AGGCTGGAGCTGCTTCG<br>**R**: ACGCGAGCCACAGGAACCGGTAGCCAGGCGCGAATCGACA ATTCCGGGGATCCGTCGAC
Plasmid	
pET22b::OmpR	**F:** ATCGCATATGCAAGAGAATTATAAGATTC<br>**R:** ATCGCTCGAGTGCTTTAGAACCGTCCG
pET22b::PhoP	**F:** ATCGCATATGCGCGTACTGGTTGTAGAGGA<br>**R:** ATCGCTCGAGGCGCAATTCAAAAAGATATC
pET22b::RpoA	**F:** CATATGCAGGGTTCTGTGACAGAGTTTCTA<br>**R:** CTCGAGCTCGTCAGCGATGCTTGCC
pGEX6p::EnvZc	**F:** ATCGGGATCCGCAGCCGGCGTGAAGCAATT<br>**R:** ATCGCTCGAGTTATGCCTCTTTTGTCGTCC
pGEX6p::FruR	**F:** CGCGGATCCAAACTGGATGAAATCGCTCG<br>**R:** CCGCTCGAGTTAGCTACGGCTCAGAATGCC
pGEX6p::GalS	**F:** CGCGGATCCATCACCATTCGTGATGTAGC<br>**R:** CCGCTCGAGTCAGTTAGTGATCAGTACCGCATTC
pGEX6p::GntR	**F:** CGCGGATCCATGAAAAAGAAAAGACCCGTACTTCAG<br>**R:** CCGCTCGAGCTA AATAGATCCGCCCGGTG
pGEX6p::NarL	**F:** ATCGGGATCCAATAATCAGGAACCGGCAACC<br>**R:** ATCGCTCGAGTTAAAAGATGCGTTCCTGATGTAC
pGEX6p::OmpR	**F:** CGCGGATCCATGCAAGAGAATTATAAGATTCTGGTGG<br>**R:** CCGCTCGAGTCATGCTTTAGAACCGTCCGGTAC
pGEX6p::PhoP	**F:** CGCGGATCCATGATGCGCGTACTGGTTGTAG<br>**R:** CCGCTCGAGTTAGCGCAATTCAAAAAGATATCCTTGTC
pGEX6p::PdhR	**F:** CGCGGATCCATGGCCTACAGCAAAATCCG<br>**R:** CCGCTCGAGCTAATTCTTGCGCTGTTCCAGGC
pGEX6p::RpoA	**F:** GGATCCCAGGGTTCTGTGACAGAGTTTCTAA<br>**R:** CTCGAGCTCGTCAGCGATGCTTGCC
pGEX6p::SsrB	**F:** GGATCCAAAGAATATAAGATCTTATTAG<br>**R:** GAATTCCATACTCTATTAACCTCATTC
pGEX6p::YjfQ	F: CGCGGATCCATGACTGAAGCACAAAGACATCAA<br>R: CCGCTCGAGTTAAACGCGGAGTATGCTTACACC
pKNT25::FruR	**F:** AACTGCAGGTGAAACTGGATGAAATCGCTCG<br>**R:** CGGAATTCTTAGCTACGGCTCAGAATGCC
pKNT25::GalS	**F:** AACTGCAGATGATCACCATTCGTGATGTAGC<br>**R:** CGGAATTCTCAGTTAGTGATCAGTACCGCATTC
pKNT25::GntR	**F:** AACTGCAGATGAAAAAGAAAAGACCCGTACTTCAG<br>**R:** CGGAATTCCTA AATAGATCCGCCCGGTG

pKNT25::OmpR	**F:** AACTGCAGATGCAAGAGAATTATAAGATTCTGGTGG<br>**R:** CGGAATTCTCATGCTTTAGAACCGTCCGGTAC
pKNT25::PhoP	**F:** AACTGCAGATGATGCGCGTACTGGTTGTAG<br>**R:** CGGAATTCTTAGCGCAATTCAAAAAGATATCCTTGTC
pKNT25::PdhR	**F:** AACTGCAGATGGCCTACAGCAAAATCCG<br>**R:** CGGAATTCCTAATTCTTGCGCTGTTCCAGGC
pKNT25::YjfQ	**F:** AACTGCAGATGACTGAAGCACAAAGACATCAA<br>**R:** CGGAATTCTTAAACGCGGAGTATGCTTACACC
pKT25::OmpR	**F:** ATCGTCTAGATCAAGAGAATTATAAGATTCT<br>**R:** ATCGGAATTCTCATGCTTTAGAACCGTCCGG
pKT25::PhoP	**F:** ATCGTCTAGATCGCGTACTGGTTGTAGAGGAT<br>**R:** ATCGGAATTCTTAGCGCAATTCAAAAAGATATC
pKT25::SsrB	**F:** ATCGTCTAGATAAAGAATATAAGATCTTATTA<br>**R:** ATCGGAATTCTTAATACTCTATTAACCTC
pKT25::RpoA	**F:** TCTAGAGGGTTCTGTGACAGAGTTTCT<br>**R:** GGATCCTCGTCAGCGATGCTTGCCGG
pTim::*ompF*	**F:** ATCGGAATTCTATTATTTCCTTTTGAAACCAAATCT<br>**R:** ATCGCTGCAGAAACAAAGGGGTCTGCTGA
pUT18C::*trxA*	**F:** TCTAGAGAGCGATAAAATTATTCACCTG<br>**R:** GAGCTCGCCAGATTGGCGTCGAGAAAC
pFLAG::OMPR ENVZ	
1. *ompR envZ* PCR	**F:** ATCGAAGCTTACCTTTGCTGTCGATATTGCGC<br>**R:** ATCGTCTAGATTATGCCTCTTTTGTCGTCCCCTGGAC
2. FLAG insert PCR	**F:** GAGTACAGACAATG***GACTACAAAGACGATGACGACAAG***CAAGAGAATTATAAGATTCTGG<br>**R:** CCAGAATCTTATAATTCTCTTG***CTTGTCGTCATCGTCTTTGTAGTC***CATTGTCTGTACTC
pTRXA::FLAG	**F:** CCGCTCGAGCGAAGTCGGA AAACTTCTGT TCTGTTAAATG<br>**R:** CGCGGATCCTTA***CTTGTCGTCATCGTCTTTGTAGTC***CGCCAGATTGGCGTCGAGAAACT
FLAG::*ompR envZ*::Cm	**F1**: ATCGGGATCCGAAAGGGAGGTATTACCCT<br>**R1**: ATCGCTCGAGCGACTGAACTGCCAGGCGT<br><br>**F2**: ACTAGTCATGGTCCATATGAATATCC<br>**R2**: ATCGGGATCCGTGTAGGCTGGAGCTGCTTC
trxA::6His::Km	**F1**: ATCGAAGCTTAAAATGATCGCTCCGATTC<br>**R1:** ATCGTCTAGATCAGTGGTGGTGGTGGTGGTGCT<br><br>**F2**: ATTTACTAGTGTGTAGGCTGGAGCTGCTTCG<br>**R2**: ATTTACTAGTATTCCGGGGATCCGTCGA

Point mutation	
ompR D55A	**F:** CATCTCATGGTACTG***GCT***TTAATGCTGCCAG<br>**R:** CACCTGGCAGCATTAA***AGC***CAGTACCATGA

ompR A136D	**F:** TCGCAGGAAGAG***GAT***GTTATCGCGTTC<br>**R:** GAACGCGATAAC***ATC***CTCTTCCTGCGA
ompR V137D	**F:** CAGGAAGAGGCC***GAT***ATCGCGTTCGGT<br>**R:** ACCGAACGCGAT***ATC***GGCCTCTTCCTG
ompR I138D	**F:** GAAGAGGCCGTT***GAT***GCGTTCGGTAAG<br>**R:** CTTACCGAACGC***ATC***AACGGCCTCTTC
ompR S174D	**F:** AAAGCGTTAGTC***GAT***CATCCGCGCGAG<br>**R:** CTCGCGCGGATG***ATC***GACTAACGCTTT
ompR E178K	**F:** AGCCATCCGCGC***AAG***CCGCTCTCTCGC<br>**R:** GCGAGAGAGCGG***CTT***GCGCGGATGGCT
ompR R182D	**F:** GAGCCGCTCTCT***GAT***GATAAGCTGATG<br>**R:** CATCAGCTTATC***ATC***AGAGAGCGGCTC
ompR L228D	**F:** ACCGTCTGGGGC***GAT***GGCTACGTCTTT<br>**R:** AAAGACGTAGCC***ATC***GCCCCAGACGGT
ompR Y230D	**F:** TGGGGCCTGGGC***GAT***GTCTTTGTACCG<br>**R:** CGGTACAAAGAC***ATC***GCCCAGGCCCCA
trxA C33A C36A	**F:** TTCTGGGCAGAGTGG***GCC***GGGCCG***GCT***AAATGATCGCTC<br>**R:** GAGCGATCATTTT***AGC***CGGCCC***GGC***CCACTCTGCCCAGAA
trxA C33D	**F:** GATTTCTGGGCAGAGTGG***GAC***GGGCCGTGTAAAATGAT<br>**R:** ATCATTTTACACGGCCC***GTC***CCACTCTGCCCAGAAATC
trxA C36D	**F:** AGAGTGGTGCGGGCCG***GAT***AAAATGATCGCTCCG<br>**R:** CGGAGCGATCATTTT***ATC***CGGCCCGCACCACTCT
trxA R74D	**F:** ATATGGCATC***GAC***GGTATTCCGACT<br>**R:** AGTCGGAATACC***GTC***GATGCCATAT
trxA G75D	**F:** GGCATCCGC***GAT***ATTCCGACTC<br>**R:** GAGTCGGAAT***ATC***GCGGATGCC
trxA I73D I76D	**F:**<br>CGCCTAAATATGGC***GAC***CGCGGT***GAT***CCGACTCTGCTGCTG<br>**R:**<br>CAGCAGCAGAGTCGG***ATC***ACCGCG***GTC***GCCATATTTAGGCG
trxA G93D	**F:** CAACCAAAGTA***GAC***GCACTGTCTAAA<br>**R:** TTTAGACAGTGC***GTC***TACTTTGGTTG
trxA A94D	**F:** CAAAGTAGGC***GAT***CTGTCTAAAGGT<br>**R:** ACCTTTAGACAG***ATC***GCCTACTTTG
trxA A94D L95D	**F:** AACCAAAGTAGGC***GATGAT***TCTAAAGGTCAGTT<br>**R:** AACTGACCTTTAGA***ATCATC***GCCTACTTTGGTT

Real time qRT-PCR	
rpoD (cyber green)	**F:** TGCCGATGATCTGCTGCTGG<br>**R:** TTCCGGGTATTCGGCAACGG
ompF (cyber green)	**F:** GTCAACAAACCGCCAGCACG<br>**R:** GACCGGTTTCGGTCAGTGGG

mgtA (cyber green)	**F:** TGATACGCACCCGGAGGGAT<br>**R:** CGATAACGCCTGCGGCAAAC
ssaV(cyber green)	**F:** GGTATCGAGAGGGTGGCGGA<br>**R:** CCGTCCATCGCACCGAGAAA
sifA(cyber green)	**F:** TTGCGATGCGCAGGCTAACT<br>**R:** GCAAAGCAAAAGCGGACCGT
rpoD (qPCR)	**F:** GTGGCTTGCAATTCCTTGAT<br>**R:** AGCATCTGGCGAGAAATA<br>**Probe:** 6-FAM- ATAAGTTCGAATACCGTCGCG-3BHQ-1
sifA (qPCR)	**F:** AGCGAAATCGTAGACTACCCC<br>**R:** CCAGACTGAATTTTCGCTGCC<br>**Probe:** 6-FAM- CCTTTTCTTGCGCTTTCCACCCAT-3BHQ-1
ssrA (qPCR)	**F:** ATATTACGCACAACCTTGCAT<br>**R:** CCAGTGAGCGATGTAGTAACCA<br>**Probe:** 6-FAM- AAGCCGACGTCATCAACACCA-3BHQ-1
ompF (IVT&qPCR)	**F:** AACTACATGACCAGCCGTG<br>**R:** CGCCATTCTGAGAGTTAATGC<br>**Probe:** 6-FAM-ACGGTCTCTCTTTCGGTATCCAGT-3BHQ-1
Gel mobility shift assay	
pompF DNA	**F:** ATTTCCTTTTGAAACCAA<br>**R:** CATCTTTCCATTCAAACTA
ompF(-)	**F:** AAACGTTAGTTTGAATGGA<br>**R:** ATAAAACTTTACAGAAAT

* Restriction enzyme sites are underlined.

** Point mutation sites are indicated in bold and italic.

*** IVT: *in vitro* transcription

Supplementary References.

1. G. E. Crooks, G. Hon, J. M. Chandonia, S. E. Brenner, WebLogo: a sequence logo generator. *Genome Res* **14**, 1188-1190 (2004).
2. S. C. Potter *et al.*, HMMER web server: 2018 update. *Nucleic Acids Res* **46**, W200-W204 (2018).
3. F. Sievers *et al.*, Fast, scalable generation of high-quality protein multiple sequence alignments using Clustal Omega. *Mol Syst Biol* **7**, 539 (2011).
4. S. Sadotra *et al.*, Structural basis for promoter DNA recognition by the response regulator OmpR. *J Struct Biol* **213**, 107638 (2021).
5. M. Song, J. S. Kim, L. Liu, M. Husain, A. Vazquez-Torres, Antioxidant Defense by Thioredoxin Can Occur Independently of Canonical Thiol-Disulfide Oxidoreductase Enzymatic Activity. *Cell Rep* **14**, 2901-2911 (2016).
6. D. Hanahan, Studies on transformation of Escherichia coli with plasmids. *J Mol Biol* **166**, 557-580 (1983).
7. M. Husain *et al.*, Redox sensor SsrB Cys203 enhances Salmonella fitness against nitric oxide generated in the host immune response to oral infection. *Proc Natl Acad Sci U S A* **107**, 14396-14401 (2010).
8. J. S. Kim *et al.*, DksA-DnaJ redox interactions provide a signal for the activation of bacterial RNA polymerase. *Proc Natl Acad Sci U S A* **115**, E11780-E11789 (2018).
9. K. A. Datsenko, B. L. Wanner, One-step inactivation of chromosomal genes in Escherichia coli K-12 using PCR products. *Proc Natl Acad Sci U S A* **97**, 6640-6645 (2000).
10. T. Tapscott *et al.*, Guanosine tetraphosphate relieves the negative regulation of Salmonella pathogenicity island-2 gene transcription exerted by the AT-rich ssrA discriminator region. *Sci Rep* **8**, 9465 (2018).
11. R. F. Wang, S. R. Kushner, Construction of versatile low-copy-number vectors for cloning, sequencing and gene expression in Escherichia coli. *Gene* **100**, 195-199 (1991).
