# Supplementary material for: Promiscuity of response regulators for thioredoxin steers bacterial virulence
Source: Nat Commun. 2022 Oct 20;13:6210. doi: 10.1038/s41467-022-33983-6 (PMC9584953; doi:10.1038/s41467-022-33983-6)
Supplement: Supplementary file 3 — Reporting Summary [file 41467_2022_33983_MOESM3_ESM.pdf]

nature portfolio

Corresponding author(s): Andres Vazquez-Torres

Last updated by author(s): 2022/05/10

Reporting Summary

Nature Portfolio wishes to improve the reproducibility of the work that we publish. This form provides structure for consistency and transparency in reporting. For further information on Nature Portfolio policies, see our Editorial Policies and the Editorial Policy Checklist.

Statistics

For all statistical analyses, confirm that the following items are present in the figure legend, table legend, main text, or Methods section.

n/a	Confirmed	
☐	☒	The exact sample size (*n*) for each experimental group/condition, given as a discrete number and unit of measurement
☐	☒	A statement on whether measurements were taken from distinct samples or whether the same sample was measured repeatedly
☐	☒	The statistical test(s) used AND whether they are one- or two-sided<br>*Only common tests should be described solely by name; describe more complex techniques in the Methods section.*
☐	☒	A description of all covariates tested
☐	☒	A description of any assumptions or corrections, such as tests of normality and adjustment for multiple comparisons
☐	☒	A full description of the statistical parameters including central tendency (e.g. means) or other basic estimates (e.g. regression coefficient) AND variation (e.g. standard deviation) or associated estimates of uncertainty (e.g. confidence intervals)
☐	☒	For null hypothesis testing, the test statistic (e.g. *F*, *t*, *r*) with confidence intervals, effect sizes, degrees of freedom and *P* value noted<br>*Give P values as exact values whenever suitable.*
☐	☒	For Bayesian analysis, information on the choice of priors and Markov chain Monte Carlo settings
☐	☒	For hierarchical and complex designs, identification of the appropriate level for tests and full reporting of outcomes
☒	☐	Estimates of effect sizes (e.g. Cohen's *d*, Pearson's *r*), indicating how they were calculated

Our web collection on statistics for biologists contains articles on many of the points above.

Software and code

Policy information about availability of computer code

Data collection	MS data were collected with an LTQ-Orbitrap model Velos Pro mass spectrometer (Thermo Fisher Scientific, Grand Island, NY); Immunoblots were visualized with the G:BOX imaging system (SYNGENE, Frederick MD); NMR spectra were collected with triple-resonance Varian 600 and 900 MHz instruments (Agilent Technologies, Santa Clara, CA); CD spectroscopy was collected in a Jasco 815 spectrometer (Jasco, Easton, MD); Autophosphorylation and EMSAs were collected with phosphorimager (Amersham, Buckinghamshire, England); Immunoblots were collected in ChemiDoc XRS imaging system (Bio-Rad, Hercules, CA); Microscale Thermonphoresis was collected on a Monolith NT.115 (Nanotemper Inc., Munchen, DE); Data for transcriptional analyses were acquired with a CFX Connect Real Time system (Biorad, Hercules, CA);
Data analysis	Data were analyzed with GraphPad Software version 9.3; MS data were analyzed with the Mascot and Protein Prospector Program (Matrix Science, Boston, MA); NMR data were analyzed with NMRPipe version 10.9 (NIST IBBR) and CCPNMR version 2.4.2, and secondary structure was determined with Secondary Structural Propensity (SSP) score program; consensus logos were analyzed with 3D-Blast (http://3d-blast.life.nctu.edu.tw/), Clustal Omega (https://www.ebi.ac.uk/Tools/msa/clustalo/) and WebLogo (https://weblogo.berkeley.edu/); Microscales thermophoresis data were analyzed with MO Affinity Analysis package (Nanotechnologies, Munchen, DE); Protein density was analyzed with ImageJ program (NIH); kd values for CD analysis were calculated with Microsoft Excel version 16.65; Images were analyzed with Image J version 1.53e (NIH, Bethesda, MD);

For manuscripts utilizing custom algorithms or software that are central to the research but not yet described in published literature, software must be made available to editors and reviewers. We strongly encourage code deposition in a community repository (e.g. GitHub). See the Nature Portfolio guidelines for submitting code & software for further information.

Data

Policy information about availability of data

All manuscripts must include a data availability statement. This statement should provide the following information, where applicable:

- Accession codes, unique identifiers, or web links for publicly available datasets
- A description of any restrictions on data availability
- For clinical datasets or third party data, please ensure that the statement adheres to our policy

All data generated or analysed during this study are included in this published article (and its supplementary information files). The NMR chemical shifts have been deposited in the Biological Magnetic Resonance Bank under accession codes 51150 (https://bmrb.io/search/instant.php?term=51150) and 51151 (https://bmrb.io/data_library/summary/index.php?bmrbId=51151). TrxA and OmpR were modeled from PDB 4HUA(https://www.rcsb.org/structure/4HUA), and (https://alphafold.ebi.ac.uk/entry/P0AA19) and PDB 6LXN (https://www.rcsb.org/structure/6lxn), respectively. The mass spectrometry proteomics data have been deposited to the ProteomeXchange Consortium via the PRIDE partner repository with the dataset identifier PXD037199 and 10.6019/PXD037199".

Human research participants

Policy information about studies involving human research participants and Sex and Gender in Research.

Reporting on sex and gender	Not applicable
Population characteristics	Not applicable
Recruitment	Not applicable
Ethics oversight	Not applicable

Note that full information on the approval of the study protocol must also be provided in the manuscript.

Field-specific reporting

Please select the one below that is the best fit for your research. If you are not sure, read the appropriate sections before making your selection.

☒ Life sciences ☐ Behavioural & social sciences ☐ Ecological, evolutionary & environmental sciences

For a reference copy of the document with all sections, see nature.com/documents/nr-reporting-summary-flat.pdf

Life sciences study design

All studies must disclose on these points even when the disclosure is negative.

Sample size	Sample size for animal studies was determined taking into account a power of 80%, alpha value of 0.05 and beta value of 0.95. No statistical package was used to determined sample size for all other experiments. With the exception of some of the CD data that was done in duplicate, all others experiments were done with a minimum of 3 biological replicates.
Data exclusions	No data were excluded from our analyses.
Replication	Experiments were repeated on 2 independent days at a minimum. Once the protocols had been troubleshooted, all replicates were included in all experiments.<br>Whenever possible, phenotypes were confirmed by two or more independent approaches.
Randomization	Mice were assigned at random to any given group. Both sexes were used. Biochemical reactions as well as host cell and microbiological cultures were randomized during collection in order to minimize extrinsic effects. NMR analyses are done with one sample at a time.
Blinding	Mouse studies were performed by Lin Liu with bacterial cultures provided unidentified by Ju-Sim KIm.<br>In most other experiments, investigators knew the identity of the specimens. However, whenever possible, key experiments were done by two individuals. The investigators new the identity of the specimens during collection of biochemical, microbiological and cell culture data , but statistical analysis was performed simultaneously on all groups within an experiment. NMR analysis cannot be done blinded, since a critical part of the analysis is based on known crystal structures.

Behavioural & social sciences study design

All studies must disclose on these points even when the disclosure is negative.

Study description	not applicable
Research sample	not applicable
Sampling strategy	not applicable
Data collection	not applicable
Timing	not applicable
Data exclusions	not applicable
Non-participation	not applicable
Randomization	not applicable

Ecological, evolutionary & environmental sciences study design

All studies must disclose on these points even when the disclosure is negative.

Study description	not applicable
Research sample	not applicable
Sampling strategy	not applicable
Data collection	not applicable
Timing and spatial scale	not applicable
Data exclusions	not applicable
Reproducibility	not applicable
Randomization	not applicable
Blinding	not applicable

Did the study involve field work? ☐ Yes ☒ No

Reporting for specific materials, systems and methods

We require information from authors about some types of materials, experimental systems and methods used in many studies. Here, indicate whether each material, system or method listed is relevant to your study. If you are not sure if a list item applies to your research, read the appropriate section before selecting a response.

Materials & experimental systems

n/a	Involved in the study	
☐	☒	Antibodies
☐	☒	Eukaryotic cell lines
☒	☐	Palaeontology and archaeology
☐	☒	Animals and other organisms
☒	☐	Clinical data
☒	☐	Dual use research of concern

Methods

n/a	Involved in the study	
☒	☐	ChIP-seq
☒	☐	Flow cytometry
☒	☐	MRI-based neuroimaging

Antibodies

Antibodies used	anti-FLAG (10 micrograms or 1:500 dilution, Catalog number F1804, Sigma-Aldrich, St Louis, MO), anti-DnaK (1:5000 dilution, cat. # ab69617, Abcam, Waltham, MA), anti- 6His (1:1000 dilution, cat. # 600-401-382, Rockland Immunochemicals, Limerick, PA), horseradish peroxidase-conjugated, goat anti-mouse IgG (1:20,000 dilution, cat. # 45-000-692, Fisher Scientific, Grand Island, NY)
Validation	Extracts isolated from Salmonella strains not expressing gene fusions of the FLAG or His epitopes were used as negative controls for the validation of anti-FLAG or anti-His antibodies by Western blotting. Salmonella strains expressing the gene of interest tagged with FLAG were used as possible controls. Western blots of cytoplasmic extracts from dnaK+ wildtype Salmonella strain ATCC 14028s and dnaK-minus DdnaK mutant strain were used to validate the anti-DnaK antibodies from Novus Biologicals.

Eukaryotic cell lines

Policy information about cell lines and Sex and Gender in Research

Cell line source(s)	J774A1 cells catalogue # TIB.67 (American Type Culture Collection, Manassas, VA)
Authentication	J774A1 cells were not authenticated beyond the testing done by ATCC.
Mycoplasma contamination	J774 cells tested nagative the with MycoAlert Mycoplasm Detection Assay at the University of Colorado School of Medicine Tissue Culture Core
Commonly misidentified lines (See ICLAC register)	J774A1 cells are not recognized by ICLAC as a commonly misidentified line.

Animals and other research organisms

Policy information about studies involving animals; ARRIVE guidelines recommended for reporting animal research, and Sex and Gender in Research

Laboratory animals	C57BL/6 mice, males and females, 6-8 week old. In house breeding from stocks obtained from Jackson labs. Mice are maintained on a 12 hour dark/light cycle, 21 degree centigrades and 40% ambient humidity.
Wild animals	No wild mice were used in the course of our investigations.
Reporting on sex	males and females were used at random
Field-collected samples	No field-collected samples were used in this study.
Ethics oversight	University of Colorado School of Medicine IACUC

Note that full information on the approval of the study protocol must also be provided in the manuscript.
